# Monitoring of potential invasive arthropod species in Azores Islands (Corvo, Flores, Faial, Pico, Terceira, São Miguel and Santa Maria): the PRIBES Project

**DOI:** 10.3897/BDJ.14.e188056

**Published:** 2026-03-23

**Authors:** Abrão Leite, Mário Boieiro, António Onofre Soares, Alejandra Ros-Prieto, Ricardo Costa, Gabor Pozsgai, Guilherme Oyarzabal, Mário Brum Teixeira, Hugo Renato Calado, Alexandra Dal Lago, Martha Vounatsi, Rosalina Gabriel, Sophie Wallon, Luís C. Crespo, Juan Pascual Gil de Gómez, Maria Teresa Ferreira, Sébastien Lhoumeau, Paulo A. V. Borges

**Affiliations:** 1 Rua Fernando Pessoa, nº99 R/C DTO 2765-483, Estoril, Portugal Rua Fernando Pessoa, nº99 R/C DTO 2765-483 Estoril Portugal; 2 University of Azores, CE3C—Centre for Ecology, Evolution and Environmental Changes, Azorean Biodiversity Group, CHANGE —Global Change and Sustainability Institute, School of Agricultural and Environmental Sciences, Rua Capitão João d’Ávila, Pico da Urze, 9700-042, Angra do Heroísmo, Azores, Portugal University of Azores, CE3C—Centre for Ecology, Evolution and Environmental Changes, Azorean Biodiversity Group, CHANGE —Global Change and Sustainability Institute, School of Agricultural and Environmental Sciences, Rua Capitão João d’Ávila, Pico da Urze, 9700-042 Angra do Heroísmo, Azores Portugal; 3 IUCN SSC Atlantic Islands Invertebrate Specialist Group, Angra do Heroísmo, Azores, Portugal IUCN SSC Atlantic Islands Invertebrate Specialist Group Angra do Heroísmo, Azores Portugal; 4 LIBRe – Laboratory for Integrative Biodiversity Research, Finnish Museum of Natural History, University of Helsinki, P.O.Box 17 (Pohjoinen Rautatiekatu 13), 00014, Helsinki, Finland LIBRe – Laboratory for Integrative Biodiversity Research, Finnish Museum of Natural History, University of Helsinki, P.O.Box 17 (Pohjoinen Rautatiekatu 13), 00014 Helsinki Finland; 5 University of Azores, CE3C—Centre for Ecology, Evolution and Environmental Changes, Azorean Biodiversity Group, CHANGE —Global Change and Sustainability Institute, Faculty of Science and Technology, Rua da Mãe de Deus, 9500-321, Ponta Delgada, São Miguel, Azores, Portugal University of Azores, CE3C—Centre for Ecology, Evolution and Environmental Changes, Azorean Biodiversity Group, CHANGE —Global Change and Sustainability Institute, Faculty of Science and Technology, Rua da Mãe de Deus, 9500-321 Ponta Delgada, São Miguel, Azores Portugal; 6 Centro de Biotecnologia dos Açores, Ponta Delgada, Azores, Portugal Centro de Biotecnologia dos Açores Ponta Delgada, Azores Portugal; 7 University of Bologna, Bologna, Italy University of Bologna Bologna Italy; 8 University of Athens, Athens, Greece University of Athens Athens Greece; 9 Doñana Biological Station - CSIC, Seville, Spain Doñana Biological Station - CSIC Seville Spain; 10 Regional Secretariat of Environment and Climate Change, Project LIFE BEETLES (LIFE 18NAT/PT/000864), Rua do Galo n118, 9700-040, Angra do Heroismo, Azores, Portugal Regional Secretariat of Environment and Climate Change, Project LIFE BEETLES (LIFE 18NAT/PT/000864), Rua do Galo n118, 9700-040 Angra do Heroismo, Azores Portugal; 11 IUCN SSC Monitoring Specialist Group, Angra do Heroísmo, Azores, Portugal IUCN SSC Monitoring Specialist Group Angra do Heroísmo, Azores Portugal

**Keywords:** Oceanic islands, exotic arthropods, alien species, biosecurity, early detection, coastal ruderal habitats, plant beating, island biodiversity, anthropogenic disturbance

## Abstract

**Background:**

Arthropods provide essential ecosystem services, yet multiple lines of evidence indicate widespread declines driven by habitat loss (degradation, fragmentation and reduction), biological invasions and climate change. Oceanic islands are particularly vulnerable to invasive alien species because of their isolation, small area and sensitivity to novel predators, competitors and pathogens. In the Azores, historical land-use change has greatly reduced native forest cover, while long-term monitoring indicates that introduced arthropod diversity is increasing even where total richness appears stable. However, ruderal coastal habitats (i.e. transitional, frequently disturbed environments often dominated by opportunistic exotic plants) remain comparatively under-sampled and may function as early “gateways” for new arthropod introductions. The PRIBES project intends to contribute to "The Regional Strategy for the Management of Terrestrial and Freshwater Exotic and Invasive Species in the Azores" (PRIBES-LIFE-IP- Estratégia regional para o controlo e prevenção de espécies exóticas invasoras - no âmbito do projeto LIFE IP AZORES NATURA, LIFE17 IPE/PT/000010). The PRIBES project addresses this gap by surveying arthropod assemblages associated with vascular plants in disturbed coastal ruderal habitats across multiple Azorean islands (Corvo, Flores, Faial, Pico, Terceira, São Miguel and Santa Maria) using a standardised time-based plant beating protocol, enabling comparisons of richness and colonisation status (endemic, native or exotic) amongst islands and vegetation contexts.

**New information:**

This manuscript provides a standardised, multi-island synthesis of arthropod sampling across seven Azorean islands, encompassing 78 sites sampled with standard methods plus one site with ad hoc samples and 23,547 specimens. It reports 366 taxa, including 247 taxa identified to species/subspecies and 119 not identified morphospecies, delivering an unusually comprehensive archipelago-scale baseline for ruderal and edge-associated assemblages. The substantial unidentified morphospecies fraction, plausibly dominated by as-yet-unrecorded Azorean arrivals despite extensive local expertise, is consistent with documented increases in island exotic arthropod diversity and highlights an identification bottleneck where recent introductions and potential pests accumulate. The study also provides major distributional updates, including 62 new island records and one new record for the Azores, corresponding to the theridiid spider *Dipoena melanogaster* (C. L. Koch, 1837). By summarising colonisation status for all identified taxa, we show a strong contribution of introduced taxa (121 of 247 identified taxa) alongside endemic (37) and native non-endemic (72) components, offering a clear quantitative snapshot of assemblage structure relevant to biosecurity and conservation planning. In addition, for 17 taxa, the colonisation status is uncertain. By publishing openly accessible, standardised occurrence records, these data directly support early detection and surveillance prioritisation for emerging introductions and help provide information for management and biosecurity strategies in rapidly changing island landscapes.

## Introduction

Arthropods are responsible for a vast number of vitally important ecological services, the most famous being pollination, but others, such as nutrient cycling via decomposition and pest control, also rely on them (Noriega et al. 2018, Schowalter et al. 2018). Despite their importance, evidence points to a decline in arthropod diversity and abundance (Cardoso et al. 2020, Wagner 2020). The driving forces of this decline are habitat reduction, degradation or fragmentation, the introduction of novel, invasive species and climate change (Borges et al. 2019, Borges et al. 2020, Pyšek et al. 2020, Stephenson et al. 2022). Studies done on continental areas of North America and Europe make up most of the evidence (Wagner 2020), yet concerns are spreading globally, especially in more remote and isolated areas (Borges et al. 2020, Lhoumeau and Borges 2023, Gerlach et al. 2024).

Oceanic islands are often remote and isolated, being known for their high concentration of endemic taxa (Gillespie and Baldwin 2014), but also the ecosystems are made more vulnerable as the balance they establish is delicate and prone to be offset by the introduction of exotics (Borges et al. 2019, Borges et al. 2020). The arrival of an exotic species, be it a predator, competitor or a pathogen, tend to heavily and negatively affect endemic arthropods (Sax and Gaines 2008, Pyšek et al. 2020). Studies carried out on islands, such as the Galápagos (Roque Albelo and Causton 1999, Causton et al. 2006), New Caledonia (Grandcolas et al. 2008) and Hawaii (Kraus and Duffy 2010) demonstrate how rapidly and profoundly invasive species can usurp and deregulate endemics, particularly those which have specialised niches and/or a low dispersal capacity.

The Azores, being an archipelago settled since the 15^th^ century, has seen most of its native forest habitat reduced to a mere 5% of its original range (Gaspar et al. 2008); thus, concerns for its remaining native biota are well founded (Borges et al. 2019). In place of the native vegetation, pastures, urban areas and exotic tree plantations now take up most of the land (Triantis et al. 2010, Rego et al. 2015).

There is an urgent need to perform long-term ecological monitoring on islands (Borges et al. 2018), particularly targeting key entry points of exotic species arrival in the archipelago, such as ports and airports. Long-term ecological monitoring programmes in Azorean islands have been instrumental in assessing the distribution and abundance of the remaining endemic and native species (Borges et al. 2020, Pozsgai et al. 2024), as well as the presence of introduced arthropods in forest habitats (Borges et al. 2022). From the long-term ecological arthropod monitoring study in Azorean Islands SLAM project (Long Term Ecological Study of the Impacts of Climate Change in the natural forest of Azores) (Borges et al. 2022a, Lhoumeau et al. 2022, Lhoumeau and Borges 2023), a common trend has emerged: while the overall species richness tends to remain stable, the diversiy of exotic species is increasing (Borges et al. 2020, Lhoumeau et al. 2022, Lhoumeau and Borges 2023).

Despite the availability of extensive data from Azorean native forests (Pozsgai et al. 2024), several anthropogenic land-use types — such as coastal urban gardens (Lamelas-Lopez et al. 2023, Borges et al. 2025) and other disturbed habitats including ruderal and coastal coastal disturbed areas — remain poorly studied (Cardoso et al. 2009, Picanço et al. 2017, Boieiro et al. 2025a, Boieiro et al. 2025b, Calado et al. 2025). These areas are often transitional zones, influenced by urban infrastructure, agriculture and transportation networks, which serve as a source of exotic species that then colonise native habitats (Borges et al. 2008, Lhoumeau et al. 2025a). Ruderal coastal habitats are often dominated by opportunistic exotic plant species and are repeatedly disturbed by human activities (e.g. trampling, clearing and infrastructure maintenance) (Chiuffo et al. 2018). Being better adapted to disturbance, exotic species also tend to colonise these areas first, given their proximity to urban zones. The majority of these exotic arthropods are thought to be accidental introductions, such as the true bug *Leptoglossus occidentalis* Heidemann, 1910 from 2024 (Van der Heyden 2024), but also others belonging to a variety of groups (see, for example, Borges et al. (2013), Borges et al. (2022a), Lhoumeau et al. (2022), Boieiro et al. (2023), Boieiro et al. (2024)).

## General description

### Purpose

This study aims to address gaps in knowledge by surveying arthropod assemblages associated with vascular plants in ruderal coastal habitats across several Azorean islands (Corvo, Flores, Pico, Faial, Terceira, São Miguel and Santa Maria). Using standardised sampling methods (time-based plant beating), we evaluate patterns of species richness, colonisation status (endemic, native, exotic) and their associations with vegetation types. We are particularly interested to answer the following questions:

-Are ruderal coastal habitats acting as gateways for the arrival and establishment of new exotic arthropods in the Azores?

-Can any endemic arthropod species persist in these highly disturbed and human-modified environments?

### Additional information

By focusing on an under-studied, under-sampled, yet widespread and mutable habitat, this study aims to contribute to a better understanding of how human disturbance, land use and biotic homogenisation affect island biodiversity. In line with similar works on other oceanic islands, these ecosystems are included in regional biodiversity networks as novel management areas. Our goal is to detect exotic species early in their expansion and propose conservation strategies to deal with the issue.

## Project description

### Title

The PRIBES Project: A survey of exotic arthropods in Azorean disturbed coastal ruderal habitats

### Personnel

Principal investigator: Paulo A. V. Borges.

Fieldwork (site selection and experimental setting): Paulo A.V. Borges.

Fieldwork (authorisation): Azorean Regional Directorate for the Environment (Internationally Recognised Compliance Certificate): 30/2020/DRCT; 58/2020/DRA; 33/2021/DRCTD; 54/2021/DRAAC; 28/2022/DRCT; 46/2022/DRAAC; CCIR-RAA/2024/6; CCIR-RAA/2025/54; LIC-106-2025-DRAAC.

Fieldwork in each Island and year:

Santa Maria (2019) - Alejandra Ros-Prieto, Paulo A. V. Borges.

Corvo (2020) - Alejandra Ros-Prieto, Maria Teresa Ferreira, Mário Boieiro, Paulo A. V. Borges; Rosalina Gabriel.

Flores (2020) - Alejandra Ros Prieto, Maria Teresa Ferreira, Mário Boieiro, Paulo A. V. Borges, Rosalina Gabriel.

Pico (2020) - Alejandra Ros Prieto, Juan Pascual Gil de Gómez, Paulo A. V. Borges, Rosalina Gabriel.

Faial (2021) - Gabor Pozsgai, Mário Boieiro, Paulo A. V. Borges, Ricardo Costa.

Terceira Island (2022, 2024, 2025) - Guilherme Oyarzabal, Martha Vounatsi, Paulo A. V. Borges.

São Miguel Island (2025) - António Onofre Soares, Hugo Renato Calado, Mário Teixeira, Paulo A. V. Borges.

Parataxonomists: Abrão Leite, Alejandro Torres Expósito, Alexandra Dal Lago, Ambre Solivellas, Amelie Neitzel, Clémence Massard, Martha Vounatsi, Noelia Reverón García, Sophie Wallon, Yerai Gómez López.

Taxonomist: Paulo A.V. Borges, Abrão Leite and Luis C. Crespo.

Database management: Paulo A. V. Borges and Sébastien Lhoumeau.

Darwin Core databases: Sébastien Lhoumeau and Paulo A.V. Borges.

### Study area description

The study area comprises seven of the nine islands (Corvo, Flores, Faial, Pico, Terceira, São Miguel and Santa Maria) within the Azores, a remote oceanic archipelago in the North Atlantic distributed along a WNW–ESE axis spanning several hundred kilometres. These islands are volcanic in origin and fall within the mid-Atlantic latitudinal band of roughly 37–40° N and 25–32° W, with the sampled islands representing the western (Corvo, Flores), central (Faial, Pico, Terceira) and eastern (São Miguel, Santa Maria) groups commonly used in biogeographic descriptions of the archipelago (Florencio et al. 2021).

**Figure 1.**
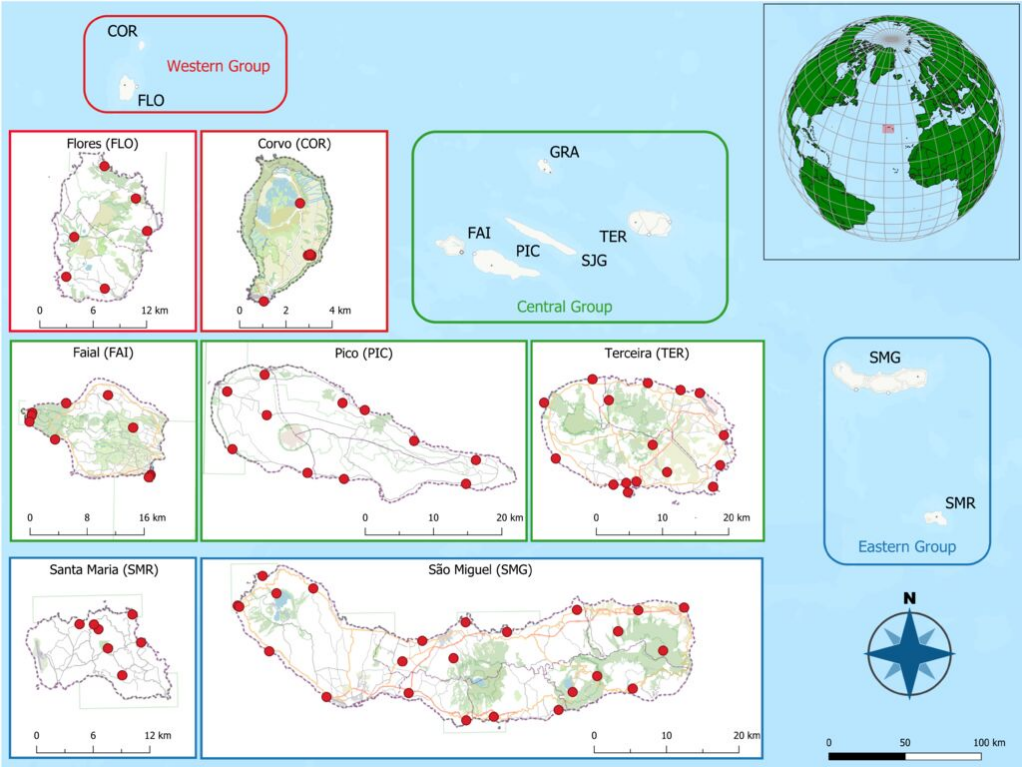
The distribution of study sites in the seven studied Azorean Islands (Credit: Gabor Pozsgai).

In our study, the archipelago is referenced by the approximate coordinate bounds 38°43′21″N 27°13′14″W and 38°27′30″N 28°19′22″W (Fig. 1), capturing the geographic envelope of the sampled islands and sites. The prevailing climate is temperate oceanic, characterised by mild conditions year-round, no pronounced dry season in most islands and strong oceanic influence on temperature and moisture regimes. Rainfall is generally regular and abundant and, together with persistent cloud cover, promotes consistently high relative humidity, often reaching very high values at mid- to high-elevation native forest sites where long-term arthropod monitoring is typically established (Borges et al. 2020, Pozsgai et al. 2024). Persistent winds are frequent, with harsher conditions commonly associated with the colder part of the year, contributing to marked seasonal differences in exposure and microclimate that can influence sampling conditions and arthropod activity (Gaspar et al. 2008).

Across the islands, land use and vegetation exhibit a strong altitudinal gradient: urban and agricultural areas are concentrated at lower elevations, extensive pasturelands and exotic plantations occupy the mid-elevations and the remaining native forests are mainly restricted to higher elevations (Gaspar et al. 2008, Florencio et al. 2021, Lhoumeau et al. 2025b).

### Design description

Sampling followed a stratified, island-wide design aimed at maximising coastal coverage while maintaining spatial independence amongst sites. Within each island, we selected multiple sampling localities that, with some few exceptions, were at least 5 km apart (Fig. 1), to reduce the likelihood of repeatedly sampling the same local arthropod pool and to improve representativeness across the coastal landscape. Site reconnaissance was conducted by travelling along the main coastal roads, complemented by the exploration of secondary roads and pedestrian paths, to ensure safe access and facilitate future re-sampling. Candidate locations were screened in the field and a site was retained only if it contained heterogeneous ruderal vegetation, including a mixture of herbaceous ruderals, shrubs and small trees, providing multiple plant architectures suitable for standardised beating.

This vegetation-based selection aligned with the project focus on disturbed coastal ruderal habitats, which are strongly influenced by human infrastructure and are expected to act as early “gateways” for the arrival of exotic arthropods. By combining a minimum-distance rule with a road-network guided search, the design balances broad spatial coverage with operational feasibility, mirroring the practical constraints typically faced by archipelago-wide monitoring programmes. In São Miguel and Terceira, we additionally included a small number of higher-elevation inland ruderal sites located along the margins of native forest fragments, to screen for early incursions of exotic arthropods potentially moving towards native habitats.

The resulting set of sites provides a consistent framework for comparing arthropod assemblages across islands and supports the project's objective of early detection and monitoring of introduced species in coastal habitats.

### Funding

Fieldwork:

-Direcção Regional do Ambiente - PRIBES (LIFE17 IPE/PT/000010) (2019-2020).

-PORBIOTA - “ACORES-01-0145-FEDER-000072 - AZORES BIOPORTAL”, funded by the Operational Programme Azores 2020 (85% ERDF and 15% regional funds) (2019-2021).

-Secretaria Regional do Ambiente e Alterações Climáticas, Project LIFE BEETLES (LIFE18 NAT/PT/0008647).

Darwin Core Database and manuscript production:

-Regional Directorate for Science, Innovation and Development [Regional Government of the Azores] through the PROSCIENTIA Incentive System (M1.1.A/FUNC.UI&D/021/2025 [UI&D/GBA/2025]).

-FCT through national and European funds by UID/00329/2025 Centre for Ecology, Evolution and Environmental Changes (CE3C) DOI https://doi.org/10.54499/UID/00329/2025.

## Sampling methods

### Sampling description

Sampling followed a standardised, time-based (1 hour) plant-beating protocol conducted along a transect across ruderal vegetation. Beating was performed by striking plant branches while holding a 1 m × 1 m beating tray beneath the foliage to collect dislodged arthropods. At each site, overall sampling effort (1 hour) was fixed (time-based); when 1–4 researchers were present, the allotted sampling time was shared amongst collectors (e.g. evenly split) and the resulting material was pooled into a single composite sample per site. Collected specimens were transferred to labelled vials and preserved in high-concentration ethanol (~ 95%) for subsequent laboratory processing and identification.

### Quality control

All scientific names in the dataset were standardised to the nomenclatural backbone provided by the most recent updated checklist of Azorean arthropods (Borges et al. 2022b), which was used as the reference source for accepted names and higher-level classification. During data curation, we checked taxa for synonymy and updated combinations, ensuring that order/family assignments and species names matched current usage before populating the Darwin Core taxonomic fields (e.g. scientificName, taxonRank, order, family). Each identified species was assigned a colonisation status according to the checklist framework (endemic, native non-endemic, introduced), allowing consistent biogeographic categorisation across islands and sampling events.

### Step description

This harmonisation ensures direct comparability of the PRIBES records with other Azorean long-term biodiversity datasets that also adopt the Borges et al. (2022b) checklist and the same colonisation-status scheme.

## Geographic coverage

### Description

The study area comprises seven of the nine islands (Corvo, Flores, Faial, Pico, Terceira, São Miguel and Santa Maria) within the Azores.

### Coordinates

36.952 and 39.709 Latitude; -31.247 and -25.048 Longitude.

## Taxonomic coverage

### Description

Phylum: Arthropoda

Class: Arachnida, Chilopoda, Diplopoda, Insecta

Order: Araneae, Archaeognatha, Blattodea, Coleoptera, Dermaptera, Hemiptera, Hymenoptera, Julida, Lepidoptera, Neuroptera, Opiliones, Orthoptera, Phasmida, Polydesmida, Psocodea, Pseudoscorpiones, Scutigeromorpha, Thysanoptera, Trichoptera.

## Traits coverage

Data on functional traits for many of the species can be found in Oyarzabal et al. (2026).

## Temporal coverage

**Data range:** 2019-9-19 – 2025-8-04.

## Collection data

### Collection name

Dalberto Teixeira Pombo (University of Azores)

### Collection identifier

DTP

### Specimen preservation method

Ethanol 95%

## Usage licence

### Usage licence

Creative Commons Public Domain Waiver (CC-Zero)

## Data resources

### Data package title

The PRIBES Project: A survey of exotic arthropods in Azorean disturbed coastal ruderal habitats

### Resource link

https://doi.org/10.15468/xch3zq

### Alternative identifiers

https://www.gbif.org/dataset/0e2398ac-2430-42dd-918a-6a2eb8533ffa

### Number of data sets

2

### Data set 1.

#### Data set name

Event Table

#### Data format

Darwin Core Archive format

#### Character set

UTF-8

#### Download URL

https://ipt.gbif.pt/ipt/resource?r=pribes_arthropods

#### Data format version

1.2

#### Description

The dataset was published in the Global Biodiversity Information Facility platform, GBIF (Borges et al. 2026). The following table include the data available for the identified species. The dataset submitted to GBIF is structured as a sample event dataset that has been published as a Darwin Core Archive (DwCA), which is a standardised format for sharing biodiversity data as a set of one or more data tables. The core data file contains 80 records (eventID). This GBIF IPT (Integrated Publishing Toolkit, Version 2.5.6) archives the data and, thus, serves as the data repository. The data and resource metadata are available for download in the Portuguese GBIF Portal IPT (Borges et al. 2026).

**Data set 1.** 

Column label	Column description
id	Unique identification code for sampling event data.
eventID	Identifier of the events, unique for the dataset.
locationID	Identifier of the location.
datasetName	The name identifying the dataset from which the record was derived.
sampleSizeValue	The numeric amount of time spent in each sampling.
sampleSizeUnit	The unit of the sample size value.
samplingEffort	The amount of effort expended during a dwc:Event.
samplingProtocol	The sampling protocol used to capture the species.
eventDate	Date or date range within which the record was collected.
day	Day of the event.
month	Month of the event.
year	Year of the event.
habitat	The habitat of the sample.
continent	Name of the continent.
stateProvince	State or province of the event.
islandGroup	Name of archipelago.
island	Name of the island.
country	Country of the sampling site.
countryCode	ISO code of the country of the sampling site.
municipality	Municipality of the sampling site.
locality	Name of the locality.
locationRemarks	Comments or notes about the dcterms:Location.
minimumElevationInMetres	The lower limit of the range of elevation (altitude, usually above sea level), in metres.
decimalLatitude	Approximate centre point decimal latitude of the field site in GPS coordinates.
decimalLongitude	Approximate centre point decimal longitude of the field site in GPS coordinates.
geodeticDatum	The ellipsoid, geodetic datum or spatial reference system (SRS) upon which the geographic coordinates given in decimalLatitude and decimalLongitude are based.
coordinateUncertaintyInMetres	Uncertainty of the coordinates of the centre of the 50 m transect in metres.
coordinatePrecision	A decimal representation of the precision of the coordinates given in the decimalLatitude and decimalLongitude.
georeferenceSources	A list (concatenated and separated) of maps, gazetteers or other resources used to georeference the Location, described specifically enough to allow anyone in the future to use the same resources.

### Data set 2.

#### Data set name

Occurrence Table

#### Data format

Darwin Core Archive format

#### Character set

UTF-8

#### Download URL

https://ipt.gbif.pt/ipt/resource?r=pribes_arthropods

#### Data format version

1.2

#### Description

The dataset was published in the Global Biodiversity Information Facility platform, GBIF (Borges et al. 2026). The following table include the data available for the identified species. The dataset submitted to GBIF is structured as an occurrence table that has been published as a Darwin Core Archive (DwCA), which is a standardised format for sharing biodiversity data as a set of one or more data tables. The core data file contains 2836 records (occurrenceID). This GBIF IPT (Integrated Publishing Toolkit, Version 2.5.6) archives the data and, thus, serves as the data repository. The data and resource metadata are available for download in the Portuguese GBIF Portal IPT (Borges et al. 2026).

**Data set 2.** 

Column label	Column description
id	Unique identification code for sampling event data.
eventID	Identifier of the events, unique for the dataset.
recordedBy	A list (concatenated and separated) of names of people, groups or organisations who performed the sampling in the field.
type	Type of the record, as defined by the Dublin Core standard.
licence	Reference to the licence under which the record is published.
institutionID	The identity of the institution publishing the data.
collectionID	The identity of the collection publishing the data.
institutionCode	The code of the institution publishing the data.
collectionCode	The code of the collection where the specimens are conserved.
datasetName	The name identifying the dataset from which the record was derived.
basisOfRecord	The nature of the data record.
occurrenceID	Identifier of the record, coded as a global unique identifier.
organismQuantity	A number or enumeration value for the quantity of organisms.
organismQuantityType	The type of quantification system used for the quantity of organisms.
sex	The sex and quantity of the individuals captured.
lifeStage	The life stage of the organisms captured.
establishmentMeans	The process of establishment of the species in the location, using a controlled vocabulary: in the GBIF database, we used 'native', 'introduced', 'endemic', "uncertain".
identifiedBy	A list (concatenated and separated) of names of people, groups or organisations who assigned the Taxon to the subject.
dateIdentified	The date on which the subject was determined as representing the Taxon.
identificationRemarks	Information about morphospecies identification (code in Dalberto Teixeira Pombo Collection).
scientificName	Complete scientific name, including author and year.
kingdom	Kingdom name.
phylum	Phylum name.
class	Class name.
order	Order name.
family	Family name.
genus	Genus name.
specificEpithet	Specific epithet.
infraspecificEpithet	Infrapecific epithet.
taxonRank	Lowest taxonomic rank of the record.
scientificNameAuthorship	Name of the author of the lowest taxon rank included in the record.

## Additional information

Across the seven sampled islands, the dataset includes 78 standard sites and 23,547 specimens, yielding 366 taxa in total, of which 247 taxa were identified to species/subspecies level and 119 were retained as morphospecies only (Table 1). Sampling effort was scaled to island area, ranging from five sites in Corvo to 24 sites in São Miguel, while specimen counts ranged from 1,406 individuals in Corvo to 5,874 in Terceira.

Taxonomic richness also varied markedly by island, with São Miguel showing the highest total taxa (194), followed by Terceira (174) and Faial (142), whereas Corvo had the lowest total taxa (80) (Table 1). A substantial fraction of the recovered diversity remained unresolved at species level, with morphospecies-only taxa ranging from 12 (Flores) to 39 (São Miguel) and representing 32% of all taxa overall (119/366) (Table 1). Considering only the identified taxa (n = 247), the community was dominated by introduced species (121 taxa; 49%), followed by native non-endemic taxa (72; 29%), endemic taxa (37; 15%) and taxa of uncertain origin (17; 7%). At the island level, the proportion of introduced taxa amongst identified species was consistently high (≈ 41–51%), peaking in Pico (51%) and São Miguel (50%), while Santa Maria showed the highest relative contribution of endemic taxa (15/74; 20%).

A tota of 62 new records are reported for individual islands (Table 1), with the largest numbers in Corvo (22) and São Miguel (16), followed by Faial (10), whereas Terceira had only one new island record. Within these new island records, most were assigned to introduced taxa (35) and native non-endemic taxa (22), while only four were endemic and one had uncertain status. The detailed list of new recorded species are available in Suppl. material 1.

Finally, only one species was recorded as new to the Azores Archipelago, the spider *Dipoena melanogaster* (C.L.Koch, 1837) (Araneae, Theridiidae), detected on São Miguel.

### Comments on relevant species

***Agyneta rugosa* Wunderlich, 1992**

*Agyneta rugosa* also known as sheet weaver spider, dwarf spider or money spider is a small spider in the Linyphiidae family. It is a rare and endangered species (Borges and Cardoso 2021), distributed across an area of only 20 km^2^. It was previously know only from the islands of Terceira, Faial, São Jorge and São Miguel (Borges et al. 2022b), now being found on Pico. These spiders often make their sheet-like webs on the ground and in between mosses on the forest floor; therefore, disturbances to the quality and size of these microhabitats threaten them. Invasive plants, that alter the forest understorey composition and topography, are especially problematic in this regard and, as it has been observed in previously known populations of this spider during the latest assessment on 26 September 2017 (Borges and Cardoso 2021), cause declining population trends of this endangered species. The finding of the new population on Pico is remarkable, but conservation measures must be taken urgently to ensure its long-term survival.

***Dipoena umbratilis* (Simon, 1873)**

A member of the comb-footed spider family (Theridiidae), this spider has been introduced to the Azorean Archipelago, specifically to the islands of Faial and Pico (Boieiro et al. 2024) and is here newly documented from Flores, Terceira and São Miguel, extending its known archipelagic distribution to five islands. It is a small, forest-dwelling species, reaching approximately 2.5 mm in length in both sexes. The body is mostly brown, with the legs more of a reddish-brown, possessing on its opisthosoma 4-5 whitish stripes. It was introduced from its native range in mainland Europe, making it important to monitor its spread and assess potential negative impacts on endangered native endemic species.

***Dipoena melanogaster* (C. L. Kock, 1837)**

*Dipoena melanogaster* (C. L. Koch, 1837) is a small theridiid (Theridiidae), originally described from the Palearctic Region, more precisely in Germany (World Spider Catalog 2025). This is a new record for Azores and was found in seven sites in São Miguel Island, which implies an already wide distrbution in the Island. Its documented global distribution spans much of Europe and extends from North Africa eastwards to Azerbaijan and Iran (World Spider Catalog 2025). European occurrence records compiled in Nentwig et al. (2025) include, amongst many others, Portugal and Spain and a broad set of central, northern and eastern European countries, consistent with a West Palearctic range. This species prefers sunny forest edges, occurring on the ground and on lower twigs of trees and bushes. In Great Britain and Ireland, it has been recorded from gorse and other low shrubs and, in Ireland, from lower branches of Scots pine, with adults reported in June–July (British Arachnological Society / Spider Recording Scheme (SRS) 2025).

***Aphrodes hamiltoni* Quartau & Borges, 2003**

**Figure 2.**
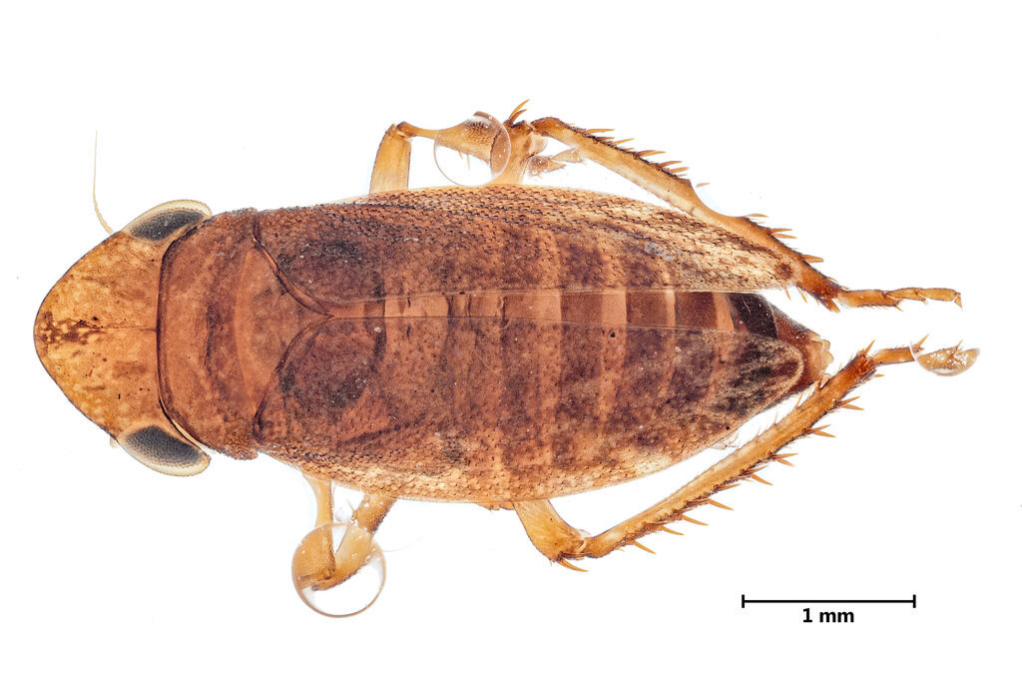
*Aphrodes hamiltoni* Quartau & Borges, 2003 (Credit: Javier Torrent, Azorean Biodiversity Group).

This leafhopper species (Cicadellidae) (Fig. 2) is endemic to the Azorean islands, being currently classified as endangered by the IUCN (Borges and Nunes 2018). It can be found in all Azorean islands, except for Corvo (Borges et al. 2022b), having an estimated area of occupancy of only 184 km², which is much smaller than its estimated extent of occurrence of 38,000 km² (Borges and Nunes 2018). This species was found in our survey for the first time to Corvo Island in a mixed patch of native and exotic tree species. The three other occurrences in the current dataset include one site in Flores and two sites in São Miguel adjacent to native forest. The species thrives mostly in the leaf litter of its native pristine forest (Quartau and Borges 2003). Unfortunately, this restricts it to fragmented patches of suitable habitat. Combined with its limited dispersal ability, at least 50% of the population is isolated in patches that are either too small to sustain viable populations long-term or are completely surrounded by non-native pastures and *Cryptomeria japonica* (L.f.) D.Don plantations. With the added pressures of invasive species, such as *Pittosporum undulatum* Vent. and *Hedychium gardnerianum* Sheppard ex Ker Gawl., as well as climate change (Ferreira et al. 2016), the species is predicted to be under severe threat in the coming decade (Borges and Nunes 2018).

***Nysius atlantidum* Horváth, 1890**

An endemic hemipteran in the Lygaeidae family already known from all islands, except for Corvo, Pico and S. Jorge (Borges et al. 2022b). The species is now recorded for the first time in Corvo Island in a coastal area. Despite the relatively small area of occupancy (AOO = 64 km²) compared to its much larger extent of occurrence (EOO = ca. 35,000 km²), it is classified as least concern due to its high abundance in native forests as well as its ability to inhabit exotic forests, demonstrating a high degree of adaptability to anthropogenic disturbances. Nevertheless, with the increased pressures of invasive species and climate change (Ferreira et al. 2016), regular population monitoring will be necessary to support decision-making on future conservation actions. It is a diurnal phytophagous species that is often found on or in the vicinity of *Erica azorica* Hochst. or herbaceous plants, near coastal habitats, occurring from sea level up to 600 m in altitude.

***Sophonia orientalis* (Matsumura, 1912)**

Commonly known as the two-spotted leafhopper, this introduced species was known from the islands of Pico and Terceira (Borges et al. 2022b), being now found for the first time on Faial and São Miguel. This is an easily identifiable leafhopper with yellowish colouration and an orange to brownish patch that accompanies a darker line running from the tip of its rostrum almost to the end of its wings. Near the wing tips, there are two dark spots that mimic eyes, providing the insect with some protection by guiding predator attacks away from the head. This species is native to Southeast Asia and is known to be polyphagous, with at least 235 recorded host plants (Aguin-Pombo et al. 2007). It is also a highly damaging species to the plants it feeds on, with symptoms including leaf distortion, total chlorosis and stunting of the plant (Jones et al. 2000). Given its expansion, known invasive potential and competition with native species for food resources (Aguin-Pombo et al. 2007), some control actions might be necessary to prevent its spread and dominance in Azorean ecosystems.

### Patterns of exotic species invasion and agricultural pests

Overall, the subset of exotic taxa found in the current survey includes: (i) an ecosystem-engineering invader (the Argentine ant, *Linepithema humile*) capable of restructuring food webs and facilitating honeydew-producing pests (Wetterer and Espadaler 2010, Angulo et al. 2024) and (ii) multiple herbivores, such as some thrips with established pest histories that can affect crops, ornamentals and potentially native vegetation through chronic plant stress and diseases. The Argentine ant *Linepithema humile* is one of the world’s best-studied invasive ants which commonly forms high-density supercolonies in invaded regions and whose spread strongly is shaped by human transport networks and “jump dispersal” (Suarez et al. 2001, Angulo et al. 2024). A recurrent mechanism relevant to agriculture is that invasive ants often protect honeydew-producing Hemiptera, increasing sap-feeder abundance and sometimes worsening plant damage or pathogen transmission risk (Schifani et al. 2024).

The exotic taxa in our survey include several polyphagous sap-feeders and cell-content feeders (e.g. *Sophonia orientalis*, *Siphanta acuta*, *Nezara viridula* and pest thrips, such as *Heliothrips haemorrhoidalis* and *Hercinothrips bicinctus*) that are widely recognised for damaging a broad spectrum of crops and ornamentals, particularly in warm or protected cultivation conditions (see, for example, Aguin-Pombo et al. (2007), Squitier (2011)). Additional plant-associated pests include specialist or crop-linked herbivores, such as the Acacia psyllid *Acizzia uncatoides* (Malumphy and Luker 2014), the flea beetle *Epitrix cucumeris* (Solanaceae-associated pest complex) (Boavida et al. 2013), the pollen beetle *Brassicogethes aeneus* (brassicas) (Bick et al. 2023) and the pea leaf weevil *Sitona lineatus* (legumes) (Vankosky et al. 2009), all of which can translate local establishment into recurrent yield/quality risks where hosts are cultivated. Forestry and woody-plant risks are exemplified by *Pissodes castaneus* (a pine-associated weevil), which can weaken stems and increase management costs where pines are planted (Skrzecz et al. 2019).

The list further includes economically important storage and structural pests, such as the rice weevil *Sitophilus oryzae* (grain storage) (Carvalho et al. 2012) and the common furniture beetle *Anobium punctatum* (seasoned timber) (Yalçın et al. 2018), which can cause chronic losses in stores and buildings even when impacts on crops are indirect.

In island settings like the Azores, these pest guilds can have disproportionately large consequences because chronic herbivory and plant stress can spill over from crops and gardens into surrounding vegetation, while pest management responses (e.g. increased pesticide use) can amplify pressures on native arthropod communities.

Taken together, the pest subset in our survey supports a clear narrative of risk concentrated in: (i) sap-feeders affecting horticulture and ornamentals; (ii) crop-linked beetles and weevils affecting brassicas/legumes/solanaceous plants and (iii) storage/wood pests affecting infrastructure and food security, all of which are priority targets for monitoring and biosecurity in insular socio-ecological systems.

As several of these taxa are already documented in Azorean monitoring datasets (including high abundances for some introduced sap-feeders including some thrips), their local relevance is not only theoretical and should be discussed in terms of surveillance and management prioritisation. In an island context, such introductions can have outsized effects because plant damage, mutualism shifts and altered habitat structure can propagate quickly into small and fragmented native ecosystems (Alyokhin et al. 2004).

### Morphospecies as sentinels of incipient invasions and pest emergence

Despite the very high taxonomic expertise of the team and the existence of a continually updated regional synthesis of the Azorean arthropod fauna (e.g. Cardoso et al. (2009), Borges et al. (2013), Borges et al. (2020), Lhoumeau et al. (2022), Borges et al. (2022a), Borges et al. (2022b), Lamelas-Lopez et al. (2023), Boieiro et al. (2023), Boieiro et al. (2024), Pozsgai et al. (2024), Calado et al. (2025)), a substantial fraction of collected individuals in broad multi-island surveys can still remain unresolved to named biological species, especially when sampling spans many orders. In our dataset, 119 taxa were retained as morphospecies, which is notable given that the Azorean checklist effort (Borges et al. 2022b) and successive long-term monitoring (e.g. Borges et al. (2022a), Lhoumeau et al. (2022), Pozsgai et al. (2024), Borges et al. (2025)) have markedly reduced identification uncertainty for much of the native/endemic fauna and for many established exotics.

**Figure 3.**
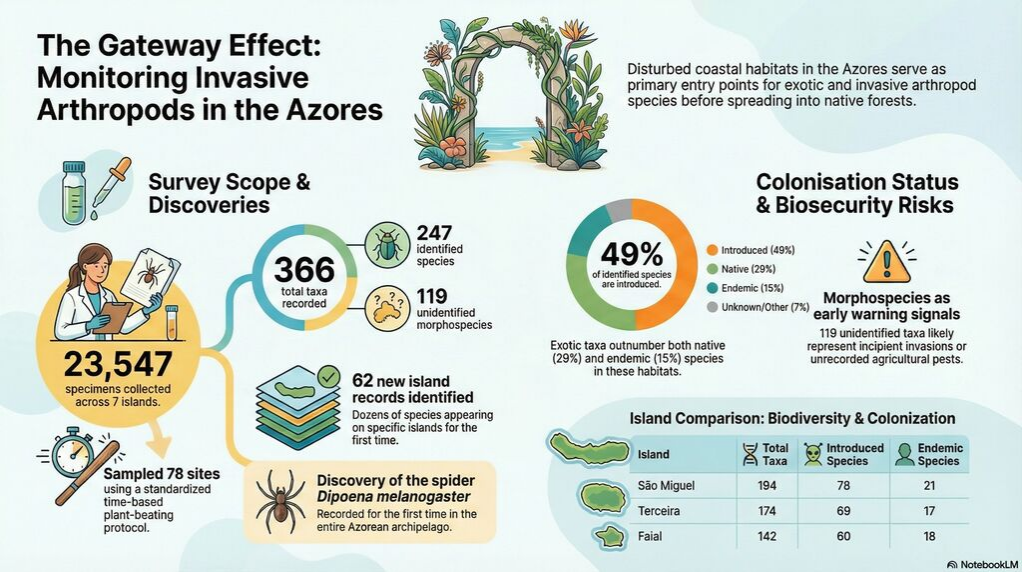
Infographic summary of the PRIBES survey of arthropods, associated with vascular plants in disturbed coastal ruderal habitats across seven Azorean islands sampled using a standardised, time-based plant-beating protocol (Credit: obtained using the IA tool NotebookLM).

Given that specimens in arthropod samples are routinely sorted as morphospecies first and then intensively worked up to species by senior taxonomists, a large residual “morphospecies tail” is best interpreted as a signal of taxa that are either newly arriving, poorly represented in reference collections or taxonomically difficult (e.g. cryptic species complexes). This interpretation aligns with island-wide evidence that exotic arthropod richness and/or relative dominance has been increasing in Azorean time series (Borges et al. 2020), meaning the system is currently in a phase where new arrivals are expected and can accumulate rapidly (Fig. 3). It is also consistent with repeated documentation of new records (often introductions) emerging from structured monitoring and inventory work in both native/mixed forests and highly human-influenced habitats across the Archipelago (Lhoumeau et al. 2022, Boieiro et al. 2023, Boieiro et al. 2024).

Ruderal/edge environments, disturbed forest habitats and urban greenspaces are particularly plausible “first-detection” arenas because they are tightly connected to propagule pressure from transport, horticulture and local human movement and they have already been shown to host rich pools of exotic arthropods in the Azores (Borges et al. 2018a, Arteaga et al. 2020, Borges et al. 2022a).

In this context, the 119 morphospecies should be treated as an early-warning component of the dataset (Fig. 3), because global evidence shows that alien introductions have not yet saturated and “emerging aliens” (species newly entering invasion pathways) are increasingly common (Seebens et al. 2017, Wyckhuys et al. 2025). From a biosecurity and applied perspective, the key implication is that a non-trivial subset of these morphospecies may include incipient invaders or agricultural pests that are present at low detectability, precisely the situation where delays in identification can undermine rapid response (Hulme 2009). This risk is amplified on islands, where habitat fragmentation and strong human modification constrain indigenous communities, while simultaneously creating multiple entry points and suitable “stepping-stone” habitats for non-native taxa (Borges et al. 2008, Borges et al. 2019).

Accordingly, a practical priority is to shorten the time from capture to reliable species-level assignment by integrating classic taxonomy with molecular workflows (DNA barcoding/reference libraries), which have a demonstrated track record in biosecurity identifications from intercepted material (Armstrong and Ball 2005). Such integration is especially relevant for morphospecies, where morphology alone often cannot deliver confident names, but sequence-based IDs can still be operational for surveillance and pathway management.

Finally, explicitly tracking morphospecies through time and across islands (e.g. persistence, spread, seasonal recurrence) can convert “unknowns” into a structured invasion-monitoring signal that complements established long-term indicators already developed for Azorean arthropod communities (see, for example, Tsafack et al. (2023)). Moreover, investigating iNaturalist records can also add new insights into species introductions (see Van der Heyden (2024), Van der Heyden (2025)).

Importantly, to move from opportunistic, project-bound snapshots to genuinely actionable biosecurity, the Azorean Government should fund a permanent, archipelago-wide early-detection programme with expanded spatial coverage and a formal cross-sector “One Health” governance (environment + agriculture + public health + veterinary services), because sustained monitoring is repeatedly identified as essential for decision-making and adaptive management (Stephenson et al. 2022), while Azorean time series and SLAM datasets already show increasing exotic dynamics and recurring new (often introduced) records that are most effectively captured through continuous, standardised surveillance rather than intermittent campaigns (Borges et al. 2020, Lhoumeau and Borges 2023).

## Supplementary Material

514ED411-7771-5F19-850F-24E50CB688A2 10.3897/BDJ.14.e188056.suppl1 Supplementary material 1 List of identified species with mention of new records Data type Excel Sheet Brief description The detailed list of identified species with the number of specimens per island. The new records for each island are marked in green. File: oo_1534887.xlsx https://binary.pensoft.net/file/1534887 Paulo A. V. Borges

## Figures and Tables

**Table 1.** Summary of sampling effort and taxonomic outcomes of the survey across the seven Azorean islands (Corvo - COR, Flores - FLO, Faial - FAI, Pico - PIC, Terceira - TER, São Miguel - SMG and Santa Maria - SMR), including number of sampled sites, total specimens and total taxa recorded per island. For each island, the table separates taxa identified to species/subspecies level from those retained as morphospecies, i.e. operational units not confidently assigned to a Linnaean species name at the time of processing. For the subset of identified taxa, the table reports the number of taxa by colonisation status (Endemic, Native non-endemic, Introduced, Uncertain, i.e. species for which we have no definitive information on its colonisation status), following the standard categorisation widely used in Azorean arthropod monitoring and aligned with the most recent checklist-based nomenclature and status assignments (Borges et al. 2022b). Island-level totals provide overall sampling effort, total diversity (including morphospecies), the colonisation-status breakdown for identified taxa and the cumulative number of new records. The new island records are based on a comparison with the last Azorean arthropod checklist (Borges et al. 2022b).

	**COR**	**FLO**	**FAI**	**PIC**	**TER**	**SMG**	**SMR**	**TOTAL**
Number of sites	5	6	9	11	16	24	7	78
Number of specimens	1406	2063	4396	3327	5874	4674	1807	23547
Total taxa	80	102	142	119	174	194	87	366
Morphospecies only	14	12	19	21	31	39	13	119
**Identified Taxa**	**66**	**90**	**123**	**98**	**143**	**155**	**74**	**247**
Endemic	7	14	18	12	17	21	15	37
Native non-endemic	29	37	40	36	49	53	22	72
Introduced	29	37	60	50	69	78	32	121
Uncertain	2	3	6	1	9	3	6	17
								
**New island records**	**22**	**5**	**10**	**4**	**1**	**16**	**4**	**62**
Endemic	2	0	0	1	0	0	1	4
Native non-endemic	10	3	3	1	0	3	2	22
Introduced	9	2	7	2	1	13	1	35
Uncertain	1	0	0	0	0	0	0	1
								
**New Records for the Azores**	**0**	**0**	**0**	**0**	**0**	**1**	**0**	**1**
